# The impact of training healthcare professionals’ communication skills on the clinical care of diabetes and hypertension: a systematic review and meta-analysis

**DOI:** 10.1186/s12875-021-01504-x

**Published:** 2021-07-15

**Authors:** Mi Yao, Xue-ying Zhou, Zhi-jie Xu, Richard Lehman, Shamil Haroon, Dawn Jackson, Kar Keung Cheng

**Affiliations:** 1 Institute of Applied Health Research, University of Birmingham, Birmingham, B15 2TT UK; 2 Department of General Practice, Peking University Health Science Center, Beijing, China; 3 Department of General Practice, Sir Run Run Shaw Hospital, Zhejiang University School of Medicine, Hangzhou, China; 4 Medical School, University of Birmingham, Birmingham, UK

**Keywords:** Diabetes, Hypertension, Communication skills, Training, Healthcare professionals

## Abstract

**Background:**

Diabetes and hypertension care require effective communication between healthcare professionals and patients. Training programs may improve the communication skills of healthcare professionals but no systematic review has examined their effectiveness at improving clinical outcomes and patient experience in the context of diabetes and hypertension care.

**Methods:**

We conducted a systematic review of randomized controlled trials to summarize the effectiveness of any type of communication skills training for healthcare professionals to improve diabetes and/or hypertension care compared to no training or usual care. We searched Medline, Embase, CINAHL, PsycINFO, Cochrane Central Register of Controlled Trials (CENTRAL), Cochrane Database of Systematic Reviews (CDSR), ClinicalTrials.gov and the World Health Organization International Clinical Trials Registry Platform from inception to August 2020 without language restrictions. Data on the country, type of healthcare setting, type of healthcare professionals, population, intervention, comparison, primary outcomes of glycated hemoglobin (HbA1c) and blood pressure, and secondary outcomes of quality of life, patient experience and understanding, medication adherence and patient-doctor relationship were extracted for each included study. Risk of bias of included studies was assessed by Cochrane risk of bias tool.

**Results:**

7011 abstracts were identified, and 19 studies met the inclusion criteria. These included a total of 21,762 patients and 785 health professionals. 13 trials investigated the effect of communication skills training in diabetes management and 6 trials in hypertension. 10 trials were at a low risk and 9 trials were at a high risk of bias. Training included motivational interviewing, patient centred care communication, cardiovascular disease risk communication, shared decision making, cultural competency training and psychological skill training. The trials found no significant effects on HbA1c (*n* = 4501, pooled mean difference -0.02 mmol/mol, 95% CI -0.10 to 0.05), systolic blood pressure (*n* = 2505, pooled mean difference -2.61 mmHg, 95% CI -9.19 to 3.97), or diastolic blood pressure (*n* = 2440, pooled mean difference -0.06 mmHg, 95% CI -3.65 to 2.45). There was uncertainty in whether training was effective at improving secondary outcomes.

**Conclusion:**

The communication skills training interventions for healthcare professionals identified in this systematic review did not improve HbA1c, BP or other relevant outcomes in patients with diabetes and hypertension. Further research is needed to methodically co-produce and evaluate communication skills training for chronic disease management with healthcare professionals and patients.

**Supplementary Information:**

The online version contains supplementary material available at 10.1186/s12875-021-01504-x.

## Introduction

Diabetes mellitus and hypertension are common chronic conditions and major risk factors for disability and mortality worldwide. It is estimated that 475 million adults were living with diabetes in 2017 and 874 million adults had systolic blood pressure of 140 mm Hg or higher in 2015 globally [1, 2]. The prevalence of diabetes and hypertension continue to increase due to aging populations and an increase in lifestyle risk factors [3, 4]. Hypertension and diabetes carry an increased risk of cardiovascular diseases, including myocardial infarction and stroke, that are among the most important causes of premature death and disability [5]. Importantly, diabetes and hypertension frequently coexist and require long-term self-management to improve outcomes and quality of life [6–8]. However, the management of hypertension and diabetes is often poor in terms of low patient awareness, poor medication compliance and incidence of preventable complications [9, 10].

Success in diabetes and hypertension care requires effective communication between health professionals and patients [11, 12]. This can enhance patient engagement and is associated with increased understanding of treatment, adherence to recommendations and patient satisfaction, as well as improved clinical outcomes [13, 14]. One systematic review of randomized trials of integrated care programs for people with type 2 diabetes found that better communication and information flow enabled timely treatment intensification, improved control of cardiometabolic risk factors and promoted self-care behaviors [15].

Effective communication skills involve active listening, empathy, the use of open questions, forming an understanding of patients’ perspectives, knowledge and expectations, and the ability to share information appropriately [16]. Healthcare professionals should be competent at acquiring and explaining relevant health information, counselling patients, providing treatment options, and building long term therapeutic relationships in order to achieve the best possible health outcomes as a core part of their skill set.

Motivational interviewing is one approach to improving communication between physicians and patients that can be used to enhance diabetes and hypertension self-care and management. Motivational interviewing is a person‐centered counseling style that enables healthcare professionals to explore patients’ motivations and facilitate behaviour change [17]. Several systematic reviews have shown that motivational interviewing is associated with improvement in self-management and glycemic control in the short-term as well as quality of life [18, 19]. A randomized trial of motivational interviewing in hypertension management suggested that it helped to sustain the clinical benefits of adherence behavior [20].

Shared decision making (SDM) is another key approach to communication that can be appropriately applied in diabetes and hypertension care [21]. SDM is defined as patients and healthcare professionals jointly discussing clinical factors, harms and benefits of treatment options and patient preferences, in order to reach a decision based on mutual agreement [22]. SDM often requires consideration of different management options, such as dietary change, exercise and medication, that may require significant lifestyle changes [23].

Despite the rising interest in improving communication skills for healthcare professionals, it remains unclear to what extent communication skills training improves the clinical management and outcomes for patients with cardiometabolic disease. This systematic review aimed to summarise the findings of randomized controlled trials on the effectiveness of communication skills training for healthcare professionals on the outcomes and experience of patients with diabetes and hypertension.

## Method

We initially conducted a scoping search for reports of any type of studies investigating the effectiveness of communication skills training for healthcare professionals on clinical and patient-reported outcomes for diabetes and hypertension care. We conducted the scoping search in EMBASE, MEDLINE, the Cochrane Central Register of Controlled Trials (CENTRAL), Cochrane Database of Systematic Reviews (CDSR), the Epistemonikos database (https://www.epistemonikos.org/), and PROSPERO (https://www.crd.york.ac.uk/PROSPERO/) using the search terms: communication, interview, shared decision making, training, diabetes and hypertension. We were unable to identify any existing or ongoing systematic reviews summarising the effectiveness of communication skills training for healthcare professionals on outcomes for patients with diabetes and hypertension. We registered our systematic review protocol on PROSPERO (registration ID: CRD42019129696) and designed and reported our review in accordance with the Preferred Reporting Items for Systematic Review and Meta-analysis Protocols (PRISMA-P) [24].

### Search strategy

The search strategy was designed (supplementary file 1) to find eligible articles reporting randomised controlled trials (RCTs) in the following databases from inception to August 2020: Medline (Ovid SP), Embase(Ovid SP), CINAHL(EBSCO Host), PsycINFO(Ovid SP),Cochrane Central Register of Controlled Trials (CENTRAL, Cochrane Library (Wiley)) and Cochrane Database of Systematic Reviews (CDSR, Cochrane Library (Wiley)). We also searched ClinicalTrials.gov (https://clinicaltrials.gov/) and the World Health Organization International Clinical Trials Registry Platform (https://www.who.int/clinical-trials-registry-platform). There was no language limitation. References from included articles were also hand searched to identify eligible studies. For ongoing or unpublished RCTs, we contacted the corresponding author by e-mail to request relevant information. Searches were documented in a table contained search term(s), information source, date of coverage and number of articles found.

### Eligibility criteria

#### Study design

All relevant RCTs, including cluster-randomised trials, were eligible for inclusion. There was no limit to the study setting and period or length of follow-up.

#### Population

Studies were eligible if they recruited healthcare professionals, including physicians, nurses, pharmacists and dietitians within primary and secondary care settings. Studies that assessed training of medical students were not included. Include studies must have assessed outcomes from adult or paediatric patients with a diagnosis of type 1 or 2 diabetes mellitus, or adults with a diagnosis of hypertension or both hypertension and diabetes. Studies that derived outcomes from patients with gestational diabetes mellitus (GDM) were not included.

#### Interventions

Eligible studies tested communication skills training, where the care of diabetes and/or hypertension was the main focus, against usual or no training as comparators. Communication skills included consultation skills, conversation, interview, and shared decision making. Studies where training was only one component in a complex intervention were not included.

#### Outcomes

Three categories of outcomes were assessed: clinical outcomes, patient reported outcomes and self-management, and measures of the patient-doctor relationship. Clinical outcomes included changes in systolic and diastolic blood pressure, body mass index (kg/m2), glycated haemoglobin (HbA1c), and lipid concentrations. Patient reported outcomes and self-management included patients’ understanding or awareness of diabetes and hypertension, risk perception, adherence to medications, self-care, quality of life, health status and wellbeing (including anxiety). The patient-doctor relationship was assessed using measures of trust, patient satisfaction and communication performance.

### Data management

All search results were uploaded into reference management software Mendeley for automatic checking of duplicate entries. Mendeley was also used to screen titles and abstracts after duplicate studies had been removed. The total number of articles before and after removal of duplicates was documented.

### Study selection

Before title and abstract screening, two reviewers (MY and XYZ) agreed on how to apply the eligibility criteria and then independently screened titles and abstracts of retrieved records according to the pre-specified eligibility criteria. Any disagreements were resolved by discussion, or when required, by a third reviewer (RL). The number of titles or abstracts selected and reasons for exclusion were recorded at all stages of the study selection process.

Full-text copies of all potentially relevant articles were retrieved and assessed independently by two reviewers for selection. Disagreements in this phase were resolved by consensus or resolved by a third reviewer. The total number of full-text articles selected and reasons for exclusion were documented.

### Data collection process

Data extraction was performed independently by two reviewers (MY and ZJX) any differences in data extraction were discussed until consensus was reached. The third reviewer (RL) helped resolve any discrepancies in the extracted data.

We extracted data onto standard Excel forms after a pilot test. Study characteristics extracted were: authors, article title, year of publication, country in which the study was performed, study design, care setting, study participants, number of participants in each intervention group, participants’ age (mean and range) and gender, eligibility criteria, details of the interventions in each trial arm, intervention duration (including the time spent on different components of training [e.g. training on theory, curriculum and content]), type of training, primary and secondary outcomes, length of follow-up, and source of funding.

For missing or unclear data, we requested further information from the first or corresponding author of the study by e-mail.

### Quality (risk of bias) assessment

We assessed the risk of bias using the Cochrane risk-of-bias tool for randomised controlled trials to classify each study as being at low, high or unclear risk of bias in each domain. The tool contains six bias domains: selection bias (random sequence generation and allocation concealment), performance bias, detection bias, attrition bias, reporting bias and other bias [25].

For cluster randomised controlled trials, we also assessed the risk of bias in terms of recruitment bias, baseline imbalance, loss of clusters, incorrect analysis and comparability with individually randomised trials, in accordance with Chapter 16.3.2 of the Cochrane Handbook for Systematic Reviews [25].

Two authors (MY and ZJX) independently assessed each trial for risk of bias. Disagreements were resolved by consensus, or by discussion with a third reviewer (RL).

### Outcomes and data synthesis

For each included study, the population, intervention, control group and outcomes were described. For binary outcomes, we calculated the relative risk (RR) and 95% confidence interval (CI) where outcomes were sufficiently reported. For continuous outcomes (e.g. Likert scales), we reported the mean difference (MD) and 95% CIs for trials that used the same or similar assessment scales. For trials that measured the same outcome with different assessment scales, we used the standardised mean difference (SMD) and 95% CIs.

We initially assessed for methodological heterogeneity by comparing studies in terms of participants, interventions, outcomes and other study characteristics. Where studies were methodologically heterogeneous, we summarized the results narratively.

Where studies were judged to be sufficiently methodologically homogeneous, we pooled their findings by meta-analysis. We investigated statistical heterogeneity between studies by considering the I^2^ statistic alongside the Chi^2^ test. Given the complex nature of training interventions we anticipated that there would be a degree of methodological heterogeneity and therefore combined study results using a random-effects model. For binary outcomes, we presented the summary estimate as a RR with a 95% CI. For continuous outcomes we presented a pooled MD or SMD with a 95%CI. All statistical analysis was conducted using Review Manager 5.3 software.

## Results

### Results of search

**Fig. 1:**
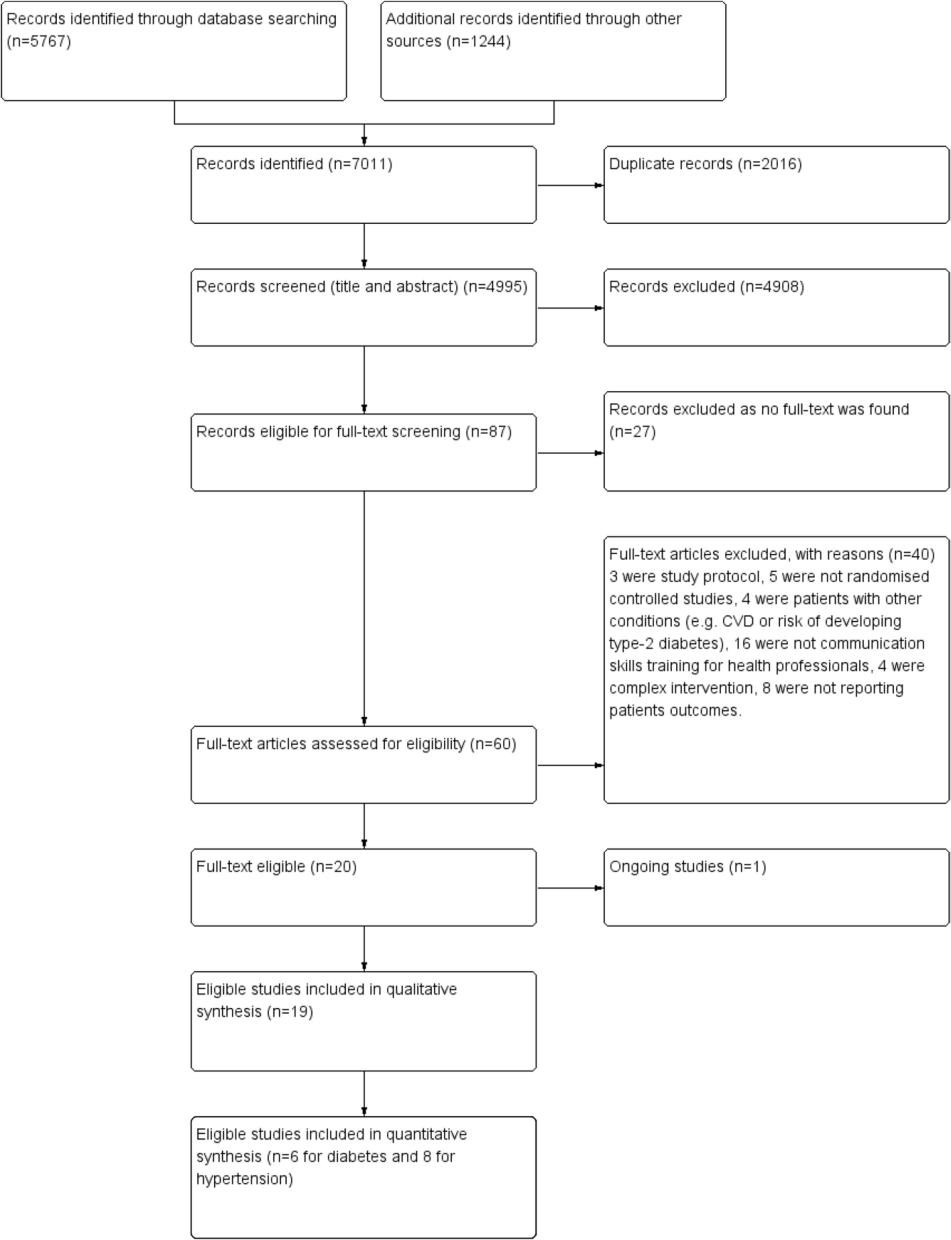
Flow diagram of training healthcare professionals in communication skills in diabetes and hypertension

7011 relevant records were identified and 4995 included in title and abstract screening after removing duplicates (Fig. 1). 87 records were eligible for full-text screening after the screening of titles and abstracts. We were unable to locate the full text for 27 articles (conference abstracts and posters). From 60 potentially eligible full-text articles, 19 original trial reports were included. 40 studies were excluded because three were study protocols, five were not randomised controlled studies, four were patients with other conditions (e.g., cardiovascular disease or at-risk of developing type-2 diabetes), 16 did not evaluate communication skills training for health professionals, four evaluated complex interventions (training did not form a significant part), and eight did not report patient outcomes.

### Characteristics of included trials

19 trials published in full were identified. 13 trials were cluster RCT (512 clusters) and 6 were individual RCT (Table 1). 21, 762 patients and 785 health professionals (484 doctors,229 nurses and 37 dietitians) were reported in these trials. 13 trials investigated the communication skills training effect on patients with diabetes: one in Type 1 DM, nine in Type 2 DM, and three in both. 6 trials investigated the training effect on patients with hypertension. 17 trials studied the effect of training on doctors and nurses, one trials for pharmacist and one for dietitians.

**Table 1:** Characteristics of included studies (n = 19, ordered by study time)

Study	Condition	Health professionals	Patients	Comparison	Outcome	Longest duration of follow-up	Country	Context	Methods
Kinmonth 1998	Type 2 diabetes	*n* = 107 (43 GPs and 64 nurses) Age: NA	*n* = 360 Mean age: 57.7 Sex: 59.2% male	Int: Patient centred care skills training Con: No training	HbA1C, blood pressure, lipids, BMI, communication performance, patient satisfaction, patient understanding, quality of life (ADDQoL), wellbing	12 months	UK	Primary care	Cluster RCT (*n* = 41)
Brug 2007	Diabetes	*n* = 37 (dietitians) Age: 24 to 45	*n* = 209 Mean age: NA Sex: NA	Int: Motivational interviewing training Con: No training	HbA1C, BMI, Self-care management	6 months	Netherlands	Home-care organizations	Individual RCT
Rubak 2009	Type 2 diabetes	*n* = 65 (GPs) Age: NA	*n* = 265 Mean age: NA Sex: NA	Int: Motivational interviewing training Con: No training	Patient-doctor relationship (Health Care Climates Questionnaire), self-care management (Summary of Diabetes Self Care Activities), patient understanding (Diabetes Illness Representation Questionnaire)	12 months	Denmark	Primary care	Cluster RCT (*n* = 48)
Sequist 2010	Diabetes	*n* = 124 (91 GPs and 33 NPs) Age: NA	*n* = 2699 Mean age: 62.4 Sex: 48.7% male	Int: Cultural competency training Con: No training	HbA1C, blood pressure, lipids, BMI, Communication performance	12 months	USA	Primary care	Cluster RCT (*n* = 31)
Heinrich 2010	Type 2 diabetes	*n* = 33 (nurses) Age: NA	*n* = 584 Mean age: 59 Sex: 45.1% male	Int: Motivational interviewing training Con: No training	HbA1C, blood pressure, lipids, BMI, patient-doctor relationship (Health Care Climates Questionnaire), self-care management (Summary of Diabetes Self Care Activities), quality of life (DSQoL)	24 months	Netherlands	Primary care	Cluster RCT (*n* = 33)
Rubak 2011	Type 2 diabetes	*n* = 140 (GPs) Age: NA	*n* = 628 Mean age: 61 Sex: 58% male	Int: Motivational interviewing training Con: No training	HbA1C, blood pressure, lipids, BMI	12 months	Denmark	Primary care	Cluster RCT (*n* = 80)
Robling 2012	Type 1 diabetes	*n* = 79 (Health practitioners) Age: NA	*n* = 693 Mean age: 4 to 15 Sex: 49% male	Int: Talking Diabetes consulting skills Con: No training	HbA1C, BMI, quality of life, self-care management	12 months	UK	Secondary and tertiary care	Cluster RCT (*n* = 26)
Farmer 2012	Type 2 diabetes	n:NA Age: NA	*n* = 211 Mean age: 63.2 Sex: 65.4% male	Int: Theory of planned behaviour training Con: No training	HbA1C Adherence (Medication Adherence Report Scale), health status (12-item Short Form Medical Outcomes), satisfaction (Diabetes Treatment Satisfaction Questionnaire)	3 months	UK	Primary care	Cluster RCT (*n* = 13)
Welschen 2012	Type 2 diabetes	n:NA Age: NA	*n* = 262 Mean age: 58.6 Sex: 43.1% male	Int: Six-step CVD risk communication training Con: No training	Patient understanding, wellbeing (Short Form Spielberger State Anxiety Inventory), risk perception (Brief Illness Perception Questionnaire), satisfaction (COMRADE scale)	3 months	Netherlands	A managed care system coordinates patients and specialists	Individual RCT
Jansink 2013	Type 2 diabetes	*n* = 53 (nurses) Age: 42.7	*n* = 521 Mean age: 64.0 Sex: 54.9% male	Int: Motivational interviewing training Con: No training	HbA1C, blood pressure, lipids, BMI, quality of life (Euroqol)	14 months	Netherlands	Primary care	Cluster RCT (*n* = 58)
Tinsel 2013	Hypertension	n:NA Age: NA	*n* = 1120 Mean age: 64.4 Sex: 45.7% male	Int: Shared decision making Con: No training	Blood pressure, patient understanding, adherence (Medication Adherence Report Scale)	20 months	Germany	Primary care	Cluster RCT (*n* = 37)
Juul 2014	Type 1 and 2 diabetes	*n* = 34 (nurses) Age: NA	*n* = 4034 Mean age: 60.5 Sex: 56.5% male	Int: Communication skills training Con: No training	HbA1C, lipids, BMI, health status (12-item Short Form Medical Outcomes), patient-doctor relationship (Health Care Climates Questionnaire), patient understanding (Problem Areas in Diabetes scale and Perceived Competence for Diabetes Scale)	18 months	Denmark	Primary care	Cluster RCT (*n* = 40)
Ma 2014	Hypertension	*n* = 12 (nurses) Age: NA	*n* = 120 Mean age: 58.8 Sex: 49.2% male	Int: Motivational interviewing training Con: No training	Blood pressure, lipids Adherence (Treatment Adherence Questionnaire of Patients with Hypertension), health status (36-item short form)	6 months	China	Primary care	Individual RCT
Manze 2015	Hypertension	*n* = 58 (doctors) Age: NA	*n* = 379 Mean age: 60.6 Sex: 29.6% male	Int: Patient-centered counseling and cultural competency training Con: No training	Blood pressure, communication performance, adherence (Hill-Bone Compliance to High Blood Pressure Therapy Scale)	18 months	USA	Primary care	Individual RCT
Kressin 2016	Hypertension	*n*:NA Age: NA	n = 8866 Mean age: 66.2 Sex: 98.8% male	Int: Patient-centered counseling Con: No training	Blood pressure, communication performance, adherence	14 months	USA	Primary care	Individual RCT
Okada 2017	Hypertension	n:NA (pharmacists) Age: NA	*n* = 125 Mean age: 64 Sex: 40% male	Int: Motivational interviewing training Con: No training	Blood pressure Adherence (Medication Adherence Report Scale), health status (WHO-Five wellbeing index and EQ-5D)	4 months	Japan	Pharmacy	Cluster RCT (*n* = 73)
Akturan 2017	Type 2 diabetes	*n* = 8 (doctors) Age: NA	*n* = 112 Mean age: 56.9 Sex: 34.8% male	Int: BATHE (Background, Affect, Troubling, Handling, and Empathy) training Con: No training	Diabetes empowerment score	6 months	Turkey	Primary care	Cluster RCT (*n* = 8)
Belin 2017	Hypertension	*n* = 35 (health providers) Age: NA	*n* = 240 Mean age: 37 Sex: 22.7% male	Int: Communication skills training Con: No training	Blood pressure, communication performance (Health Literacy Assessment Questions), adherence, patients’ self-efficacy	NA	Iran	Primary care	Individual RCT
Ismail 2018	Type 2 diabetes	n:NA (nurses) Age: NA	*n* = 334 Mean age: 58.9 Sex: 48.8% male	Int: Diabetes-6 (six psychological skill) training Con: No training	HbA1C, blood pressure, lipids, BMI, health status (PHQ-9 and Diabetes Distress Scale)	18 months	UK	Primary care	Cluster RCT (*n* = 24)

NA: not available; Int: intervention; Con: control

### Type and duration of intervention

8 trials aimed to train health professionals in motivational interviewing with theory and specific skills (Table 2). 4 trials focused on patient centered care communication training. 2 trials aimed at cultural competency training. 1 trial investigated shared decision making training and another one deployed psychological skills training. The remaining 5 trials mainly used general communication training as an intervention (e.g., risk communication, BATH interview (Background, Affect, Troubling, Handling, and Empathy), and constructive consultations). Most trials used the following methods: teaching curriculum, lectures, group discussions, workshops, role played interaction, web-based modules and feedback to implement communication skills training. The total length of training in 8 trials was more than two days, in 3 trials was less than one day. 9 trials reported training design and evaluation before study.

**Table 2:** Communication skills training of included studies (*n* = 19, ordered by study time)

Study	Conceptual frameworks or theory for interventions	Training content	Training types	Number of sessions	Training evaluation reported before trials
Kinmonth 1998	Action research	Training aimed patient centred care. The first half day was to review the evidence for patient centred consulting and a further full day was to practice skills with a facilitator, including active listening and negotiation of behavioural change.	Lectures, group discussions	1.5 days	Yes
Brug 2007	NA	Training aimed motivational interviewing (MI). The first day was to introduce MI theory and principles and the second day was to practice MI skills. Another one-day follow-up workshop for discussing experiences with experts and refresh knowledge. Training was developed and conducted by authors.	Workshop	3 days	No
Rubak 2009	NA	Training aimed motivational interviewing. A book was used to guide specific skills e.g. empowerment, ambivalence, the decisional balance schedule, the visual analogue scale, stage of change, and reflective listening. The courses consisted of a 1½-day training sessions with a half-day follow-up twice. Training was conducted by only one trained teacher.	NA	2.5 days	No
Sequist 2010	NA	Training aimed cultural competency. Training goals included understanding attitudes of trust and bias, increasing knowledge about health disparities and skills. The curriculum reviewed potential racial and cultural biases in health care, appropriate methods of collecting clinically relevant cultural data, and ways to incorporate such information into effective clinical care plans for diabetes.	Lectures, group discussions, community engagement activities	2 days	No
Heinrich 2010	NA	Training aimed motivational interviewing. Trainees received a project folder with information about the study, training material (e.g. cases for role-playing), background information about MI. Trainees received instruction charts specifying counselling techniques. Trainees were visited three times after being trained.	Role play, discussions, audio-taped consultations feedback	21.5 h	No
Rubak 2011	NA	Training aimed motivational interviewing. Training was conducted by a trained teacher. Training included specific skills, e.g. empowerment, ambivalence, the decisional balance schedule, the visual analogue scale, stage of change, and reflective listening.	NA	2.5 days	No
Robling 2012	Medical Research Council (MRC) framework	Training aimed constructive consultations (Talking Diabetes).Training emphasized shared setting of agendas and a guiding communication style, strategies and skills drawn from motivational interviewing practice.	Role play, web based modules, work shop, case studies	2 days	Yes
Farmer 2012	Theory of Planned Behaviour	Training aimed theory of planned behaviour. These included perceived benefits and harms of taking medicines. Positive beliefs were reinforced verbally and non-verbally through provision of tailored information and problem solving was facilitated around negative beliefs.	Audio-taped consultations feedback	1 day	Yes
Welschen 2012	Leventhal’s self-regulation theory,Theory of Planned Behavior	Training aimed cardiovascular disease risk communication. This included communication of the absolute risk, visual communication, message framing, communication with the patient for a reaction.	NA	1 day	No
Jansink 2013	NA	Training aimed motivational interviewing and agenda setting. This included building motivation for change, asking open questions, listening reflectively, affirming, summarizing, eliciting change, expressing empathy, developing discretion, rolling with resistance and supporting self-efficacy. Training were spread equally over 6 months.	Video recording feedback	2 days	Yes
Tinsel 2013	NA	Training aimed shared decision making (SDM) and motivational interviewing. This included risk communication, the process steps of SDM, introduction of a decision table with options.	Role play	NA	Yes
Juul 2014	Self-determination theory	Training aimed self-determination theory. This included patient-health care provider relationships, communication skills, patient worksheets, implementation of the course content in daily practice.	NA	2 days	Yes
Ma 2014	Social cognitive theory	Training aimed motivational interviewing and social cognitive theory. Training was presented by a certified trainer. Training included building rapport with the patients, evaluating the patients’ confidence and motivation for behaviour changes, helping change patients behaviours and so on.	Lectures, role play, discussions	3 days	Yes
Manze 2015	NA	Training aimed patient-centered counseling and cultural competency training. Training was led by experts in medicine and patient-centered counseling. Training includes implementing 5 A's: ask the patient about their BP management, assess their medication adherence, advise the patient about pharmacologic treatment, assist them in overcoming barriers to treatment adherence and arrange for follow-up. The cultural competency training included understanding patients, their social and financial risks for non-adherence, their fears and concerns.	Role play, work shop	2 sessions	No
Kressin 2016	NA	Training aimed patient-centered counseling. Training was led by an experienced trainer. Training includes implementing 4 A's: ask about patients’ hypertension beliefs, assess patients’ prior experiences in changing behaviors, assist patients in making needed changes, and address relapse.	Role play, discussions	2 h	Yes
Okada 2017	NA	Training aimed modified motivational interviewing. Training was based on empowerment or coaching-style communication, including: using an open question, setting each goal with patients, and closing with encouragement.	NA	4 h	No
Akturan 2017	NA	Training aimed BATHE interview (Background, Affect, Troubling, Handling, and Empathy). Training was evaluated by researchers. Trainees were asked to use the BATHE technique on their patients 3 times, with 3-month intervals.	Role play	3 h	No
Belin 2017	NA	Training aimed patient-centered counseling. Training was led by a doctor specialist. Trainees were used open-ended questions to identify the needs, barriers, patient beliefs, and ideas consistent with the patient centered counseling approach. Trainees identified that poor patient–provider communication and improved communication skills. Training was conducted using a training package and a self-assessment checklist.	Focus-group discussion, workshop	5 sessions	No
Ismail 2018	NA	Training aimed six psychological skills. The six skills were drawn from MI and CBT, including: active listening; managing resistance; directing change; supporting self-efficacy; addressing health beliefs and shaping behaviours.	NA	NA	Yes
Kinmonth 1998	Action research	Training aimed patient centred care. The first half day was to review the evidence for patient centred consulting and a further full day was to practice skills with a facilitator, including active listening and negotiation of behavioural change.	Lectures, group discussions	1.5 days	Yes
Brug 2007	NA	Training aimed motivational interviewing (MI). The first day was to introduce MI theory and principles and the second day was to practice MI skills. Another one-day follow-up workshop for discussing experiences with experts and refresh knowledge. Training was developed and conducted by authors.	Workshop	3 days	No
Rubak 2009]	NA	Training aimed motivational interviewing. A book was used to guide specific skills e.g., empowerment, ambivalence, the decisional balance schedule, the visual analogue scale, stage of change, and reflective listening. The courses consisted of a 1.5 day training sessions with a half-day follow-up twice. Training was conducted by only one trained teacher.	NA	2.5 days	No
Sequist 2010	NA	Training aimed cultural competency. Training goals included understanding attitudes of trust and bias, increasing knowledge about health disparities and skills. The curriculum reviewed potential racial and cultural biases in health care, appropriate methods of collecting clinically relevant cultural data, and ways to incorporate such information into effective clinical care plans for diabetes.	Lectures, group discussions, community engagement activities	2 days	No
Heinrich 2010	NA	Training aimed motivational interviewing. Trainees received a project folder with information about the study, training material (e.g., cases for role-playing), background information about MI. Trainees received instruction charts specifying counselling techniques. Trainees were visited three times after being trained.	Role play, discussions, audio-taped consultations feedback	21.5 h	No
Rubak 2011	NA	Training aimed motivational interviewing. Training was conducted by a trained teacher. Training included specific skills, e.g., empowerment, ambivalence, the decisional balance schedule, the visual analogue scale, stage of change, and reflective listening.	NA	2.5 days	No
Robling 2012	Medical Research Council (MRC) framework	Training aimed constructive consultations (Talking Diabetes). Training emphasized shared setting of agendas and a guiding communication style, strategies and skills drawn from motivational interviewing practice.	Role play, web-based modules, workshop, case studies	2 days	Yes
Farmer 2012	Theory of Planned Behaviour	Training aimed theory of planned behaviour. These included perceived benefits and harms of taking medicines. Positive beliefs were reinforced verbally and non-verbally through provision of tailored information and problem solving was facilitated around negative beliefs.	Audio-taped consultations feedback	1 day	Yes
Welschen 2012	Leventhal’s self-regulation theory, theory of planned behavior	Training aimed cardiovascular disease risk communication. This included communication of the absolute risk, visual communication, message framing, communication with the patient for a reaction.	NA	1 day	No
Jansink 2013	NA	Training aimed motivational interviewing and agenda setting. This included building motivation for change, asking open questions, listening reflectively, affirming, summarizing, eliciting change, expressing empathy, developing discretion, rolling with resistance, and supporting self-efficacy. Training was spread equally over 6 months.	Video recording feedback	2 days	Yes
Tinsel 2013]	NA	Training aimed shared decision making (SDM) and motivational interviewing. This included risk communication, the process steps of SDM, introduction of a decision table with options.	Role play	NA	Yes
Juul 2014	Self-determination theory	Training aimed self-determination theory. This included patient-health care provider relationships, communication skills, patient worksheets, implementation of the course content in daily practice.	NA	2 days	Yes
Ma 2014	Social cognitive theory	Training aimed motivational interviewing and social cognitive theory. Training was presented by a certified trainer. Training included building rapport with the patients, evaluating the patients’ confidence and motivation for behaviour changes, helping change patients behaviours and so on.	Lectures, role play, discussions	3 days	Yes
Manze 2015	NA	Training aimed patient-centered counseling and cultural competency training. Training was led by experts in medicine and patient-centered counseling. Training includes implementing 5 A's: ask the patient about their BP management, assess their medication adherence, advise the patient about pharmacologic treatment, assist them in overcoming barriers to treatment adherence and arrange for follow-up. The cultural competency training included understanding patients, their social and financial risks for non-adherence, their fears and concerns.	Role play, workshop	2 sessions	No
Kressin 2016	NA	Training aimed patient-centered counseling. Training was led by an experienced trainer. Training includes implementing 4 A's: ask about patients’ hypertension beliefs, assess patients’ prior experiences in changing behaviors, assist patients in making needed changes, address relapse.	Role play, discussions	2 h	Yes
Okada 2017	NA	Training aimed modified motivational interviewing. Training was based on empowerment or coaching-style communication, including using an open question, setting each goal with patients, and closing with encouragement.	NA	4 h	No
Akturan 2017	NA	Training aimed BATHE interview (Background, Affect, Troubling, Handling, and Empathy). Training was evaluated by researchers. Trainees were asked to use the BATHE technique on their patients 3 times, with 3-month intervals.	Role play	3 h	No
Belin 2017	NA	Training aimed patient-centered counseling. Training was led by a doctor specialist. Trainees were used open-ended questions to identify the needs, barriers, patient beliefs, and ideas consistent with the patient centered counseling approach. Trainees identified that poor patient–provider communication and improved communication skills. Training was conducted using a training package and a self-assessment checklist.	Focus-group discussion, workshop	5 sessions	No
Ismail 2018	NA	Training aimed six psychological skills. The six skills were drawn from MI and CBT, including: active listening; managing resistance; directing change; supporting self-efficacy; addressing health beliefs and shaping behaviours.	NA	NA	Yes

NA: not available

### Measurement of outcomes

Most trials used the following clinical outcome measures: HbA1_C_ (8 trials), blood pressure or blood pressure control (10 trials), BMI (8 trials), lipids (7 trials). Many different validated questionnaires were used to measure patients’ quality of life(5 trials), beliefs, understanding, knowledge (6 trials), self-determination, self-care, self-efficacy, empowerment, enablement, confidence (8 trials), medication adherence (7 trials), patient-doctor relationship (6 trials) and psychological well-being (4 trials). These questionnaires were:

- The diabetes specific quality of life (1 trial)
- The EuroQol (1 trial)
- Audit of diabetes dependent quality of life (1 trial)
- The EQ-5D (1 trial)
- The SF-12 (1 trial)
- Determinants of Lifestyle Behavior Questionnaire (1 trial)
- The Problem Areas in Diabetes (PAID) scale (1 trial)
- The Diabetes Empowerment Process Scale (1 trial)
- The chronic disease self-efficacy scales (1 trial)
- The Management Self Efficacy Scale for people with DM2 (1 trial)
- The Summary of Diabetes Self Care Activities (2 trials)
- The Diabetes Illness Representation Questionnaire (1 trial)
- The Brief Illness Perception Questionnaire (1 trial)
- The Perceived Competence for Diabetes Scale (1 trial)
- The Treatment Self-Regulation Questionnaire (2 trials)
- The Clinician & Group Survey – Adult Primary Care Questionnaire (1 trial)
- The Medication Adherence Report Scale (2 trials)
- The Hill-Bone Compliance to High Blood Pressure Therapy Scale (1 trial)
- The Health Care Climates Questionnaire (3 trials)
- The Patients’ perceived participation (1 trial)
- The Combined Outcome Measure for Risk communication and treatment Decision making Effectiveness scale (1 trial)
- Diabetes Treatment Satisfaction Questionnaire (1 trial)
- The Health Literacy Assessment Questions (1 trial)
- The Short Form Spielberger State Anxiety Inventory (1 trial)
- The PHQ- 9 (1 trial)
- The Diabetes Distress Scale (1 trial)

### Assessment of risk of bias in include studies

**Fig. 2:**
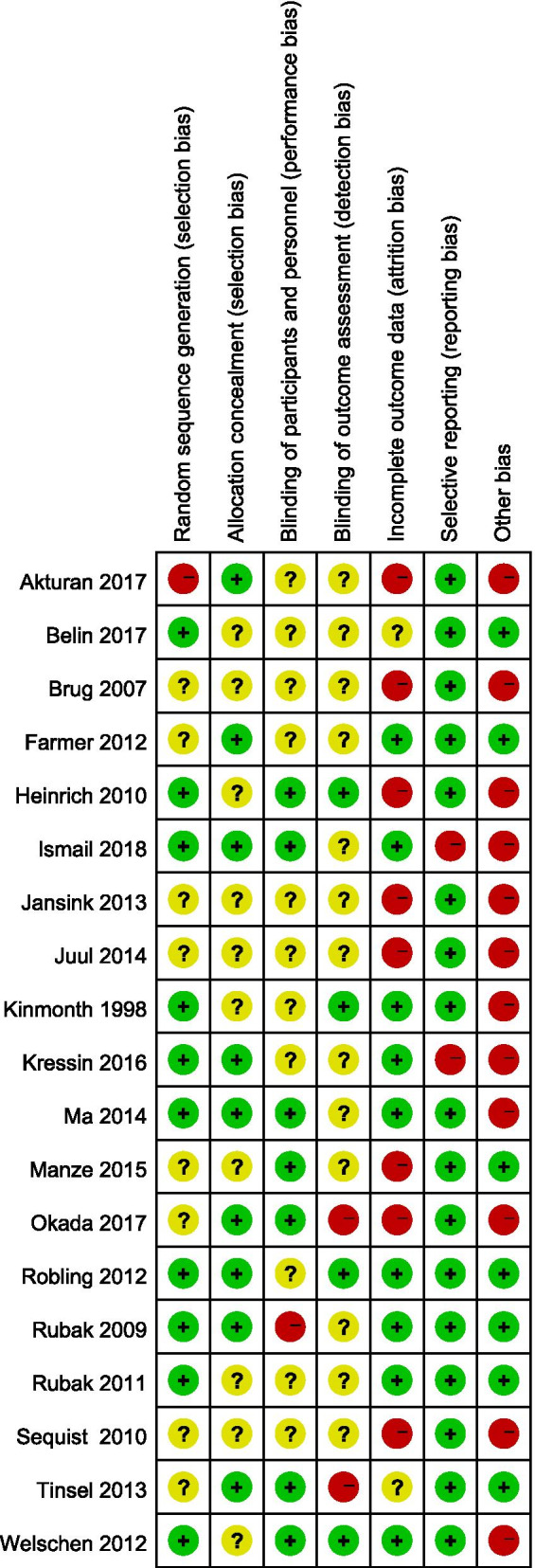
Risk of bias summary: review authors' judgements about each risk of bias item for each included study

We considered studies at a low risk of bias if they had at least 4 items (7 in total) assessed as low risk of bias. 10 trials were at a low risk and 9 trials were at a high risk of bias. See Fig. 2, 3 for the summary of all studies according to different categories of risk of bias.

**Fig. 3:**
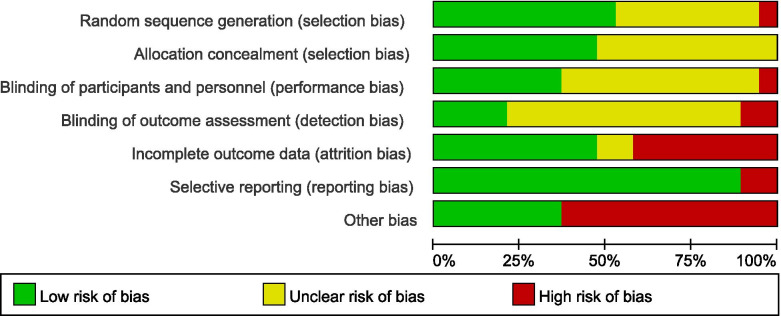
Risk of bias graph: review authors' judgements about each risk of bias item presented as percentages across all included studies

### Effectiveness of communication skills training for health professionals on clinical outcomes in patients with T2DM and hypertension

**Fig. 4:**
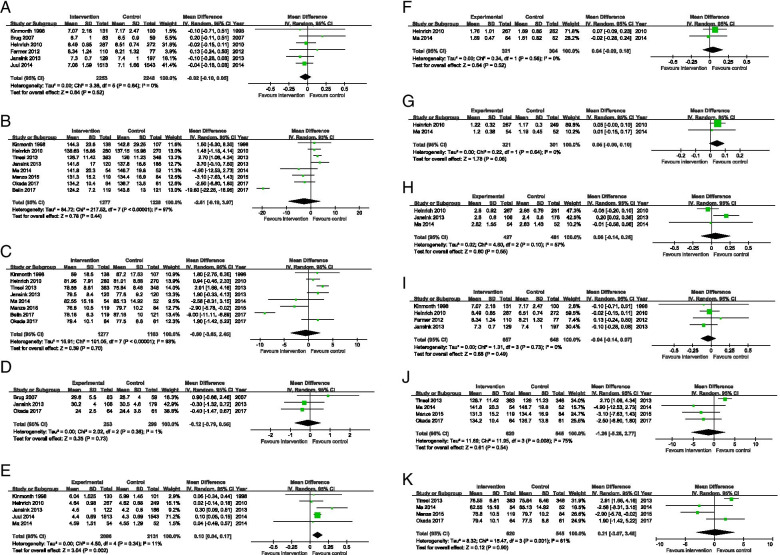
Forest plot of comparison: HbA_1_c, SBP and DBP. **A.** Forest plot of comparison: HbA_1_c. **B.** Forest plot of comparison: SBP. **C.** Forest plot of comparison: DBP. **D.** Forest plot of comparison: BMI. **E.** Forest plot of comparison: TC. **F.** Forest plot of comparison: TG. **G.** Forest plot of comparison: HDL. **H.** Forest plot of comparison: LDL. **I.** Forest plot of comparison: HbA_1_c (subgroup for T2DM studies). **J.** Forest plot of comparison: SBP (subgroup for Hypertension studies). **K.** Forest plot of comparison: DBP (subgroup for Hypertension studies)

For HbA1_C_, systolic and diastolic blood pressure, BMI, triglyceride, LDL and HDL cholesterol, there is no statistical significance at the meta-analysis level when comparing communication skills training for healthcare professionals with usual care or no training. For total cholesterol, there is a small difference at the meta-analyses level. Subgroup analysis was also conducted. (Fig. 4 and Table 3).

**Table 3:** Meta-analysis results across all outcomes

Outcomes	Studies	Number of patients	I^2^ (%)	Pooled effects (95% CI)
HbA_1c_ (%)	6	4501	0	-0.02(-0.01 to 0.05)
Systolic blood pressure (mm Hg)	8	2505	97	-2.61(-9.19 to 3.97)
Diastolic blood pressure (mm Hg)	8	2440	93	-0.60(-3.65 to 2.45)
body mass index (kg/m2)	3	552	1	-0.12(-0.79 to 0.55)
Triglyceride (mmol/L)	2	625	0	0.04(-0.09 to 0.18)
Total cholesterol (mmol/L)	5	4217	11	0.10(0.04 to 0.17)
LDL cholesterol (mmol/L)	3	908	57	0.06(-0.14 to 0.26)
HDL cholesterol (mmol/L)	2	622	0	0.05(-0.00 to 0.10)

### Effectiveness of communication skills training for health professionals on patients report outcomes

#### Quality of life

Four studies reported on quality of life. Three studies (Heinrich 2010, Jansink 2013, Okada 2017) found no difference between groups and one study (Robling 2012) found a small improvement in the control group compared with the intervention group.

#### Beliefs, understanding, knowledge

Six studies reported on patients’ understanding and living with conditions. Three studies (Rubak 2009, Heinrich 2010, Welschen 2012) found significantly better understanding and higher knowledge-scores in intervention group compared to the control group. However, one study (Welschen 2012) found that this effect was lost as time went on. Two studies (Tinsel 2013, Okada 2017) found no differences between groups. Another one study (Kinmonth 1998) found that the intervention group’s knowledge scores were lower than in the control group.

#### Self-determined, self-care, self-efficacy, empowerment, enablement and confidence

Eight studies reported on patients’ self-care and empowerment. Four studies (Rubak 2009, Robling 2012, Belin 2017, Akturan 2017) found significant evidence in the intervention group. Four studies (Heinrich 2010, Tinsel 2013, Ma 2014, Juul 2014) found no difference between groups.

#### Medication adherence

Five studies reported on medication adherence. Two studies (Ma 2014, Belin2017) found significant evidence in the intervention group while two studies (Rubak 2011, Tinsel 2013, Manze 2015) did not find any differences between groups.

#### Patient-doctor relationship

Six studies reported on patient-doctor relationship. Three studies (Rubak 2009, Heinrich2010, Juul 2014) used Health Care Climates Questionnaire as a measurement and one study (Farmer 2012) showed that there was no difference between groups. Two studies (Welschen 2012, Kinmonth 1998) found significant improvement in the intervention groups.

## Discussion

19 eligible studies were selected from 7011 potentially relevant records in this systematic review. Within these studies, a total of 21,762 patients and 785 health professionals were recruited. 13 trials investigated the communication skills training effect in diabetes and 6 trials in hypertension. There was a great clinical and methodological heterogeneity of studies in terms of training type and outcomes measurement. For the assessment of risk of bias in included studies, nearly half of trials were at a high risk of bias. The pooled results for primary outcome of HbA1C, blood pressure, BMI, TG, LDL and HDL showed that there was no evidence of differences when comparing training with usual care or no training. It was uncertain whether training for healthcare professionals was effective in secondary outcomes, e.g., quality of life, beliefs, understanding, knowledge, self-care, self-efficacy, empowerment, medication adherence and patient-doctor relationship.

The diversity of interventions and outcome measurements might be the reasons for no obvious effect or a small effect of training healthcare professionals in communication skills in this systematic review. For the training intervention, training theory, types, trainers, training assessment and evaluation, training length (only a few hours for some training) had an impact on effectiveness. It was not clear what was used to assess trained healthcare professionals in their real-world clinical practice, although three studies (Farmer 2012, Jansink 2013, Heinrich 2010) mentioned evaluations of audiotapes of consultations. In addition, the length of follow-up ranged from 3 to 24 months, so that only short-term effects were measured in the management of these long-term conditions. For clinical indicators as outcome measurements, such as HbA1_C_, blood pressure control and lipids, our findings suggest that none of the methods used to train healthcare professionals lead to significant improvements in patients with diabetes or high blood pressure. For the secondary outcomes, more than 20 questionnaires were used in studies included in this systematic review, though the same unified questionnaire was occasionally used. This makes it impossible to make direct between-study comparisons for these endpoints.

To our knowledge, this is the first systematic review to evaluate the effectiveness of training programs in healthcare professionals in communicating with patients with common chronic conditions. Patients with diabetes and hypertension typically communicate with health professionals several times a year, over the whole of their lives following diagnosis. The quality of these encounters can be a major determinant of the quality of their long-term outcomes. This systematic review addressed the question of whether the short-term clinical outcomes of patients and patient's experience can be improved through training health providers in better communication skills.

This review shows serious limitations in the evidence needed to support the development of effective training programs for health professionals caring for patients with diabetes or high blood pressure. The interventions in the included trials are often poorly characterized and are very heterogeneous, both in content and duration. The studies span 9 countries with differing types of diabetes care and major differences of culture and patient expectation. Without clearer understanding of the baseline characteristics of each system and its decision-making professionals, it is difficult to compare or to extrapolate from one setting to another. Because of this great heterogeneity among studies, many patient-related outcomes could not be compared directly.

The results of our study are similar with previous studies in other clinical areas. Although sufficient evidence is lacking, some of the included studies show a small effect on patients' understanding, self-care, and doctor-patient relationships. In a systematic review on communication skills training for healthcare professionals in cancer patients, communication training programs improve some types of healthcare professionals skills related to information gathering and supportive skills. However, the sustained effects of intervention were unable to determine over time. Also, the types of communication skills training courses evaluated in these trials were diverse. They found no evidence for the beneficial of intervention in patient’s mental or physical health, and patient satisfaction [45]. One systematic review on training clinicians on patients in primary care and rehabilitation settings found it has a small effect in improving patients satisfaction [46]. Most of communication training they found emphasized patient participation. Theoretical workshops, written information, and discussion sessions with audiovisual resources were used as communication training methods. The number of intervention sessions given by trained people varied from 1 to 12 within 1 day to 6 months. In another systematic review on communication skills training for mental health professionals in patients with severe mental illness found a modest positive effect on patient-doctor relationship. However, in this systematic review, only one pilot cluster-RCT was recruited [47]. There were relatively few good quality data and the trial is too small to highlight differences in most outcome measures, such as patient satisfaction. Similarly, previous studies show that communication skills training programs employ many different teaching theories, methods and forms of evaluation [48].

Purposeful training is a key element to the improvement of any health system, especially in systems which aim to build new capacity. This applies to the care of diabetes and hypertension in most countries, where a key aim is to maximize the potential of primary care and to encourage patient understanding and self-management. It is disappointing therefore to find that the evidence to guide such training is poor or absent. There is no generic short-term solution which will work in all contexts.

The successful management of these conditions usually depends on more than one health professional and always involves the patient. Increasingly, patients with diabetes or high blood pressure are being encouraged to self-monitor and self-manage, and to share decisions about their management. We would therefore suggest that any successful training system needs to be based on these goals, and that baseline gaps in provision and understanding need to be assessed for health professionals and patients. The key metrics would therefore be the fulfilment of these prespecified gaps in care, rather than the variety of scalar metrics which were applied across the studies in this review. Future studies should be long-term and adaptive to local need.

## Conclusion

The communication skills training interventions for healthcare professionals did not improve HbA1c, BP or other relevant outcomes in patients with diabetes and hypertension. Although the studies analyzed probably include the key ingredients for successful communication training, the timescale and format of the programs was inadequate to result in measurable change to patient-important outcomes. Better methodology is urgently needed to yield generalizable evidence for the management of these conditions of lifelong risk which affect a substantial proportion of the human population. The pooled analysis of short-term training interventions is likely to be of less value than a mixed-methods approach to training programs over longer time scales and across a range of health systems. Our study indicates that we are still some ways from identifying the methods by which patients and health professionals can reach shared understanding to achieve the best outcomes for at-risk individuals and populations.

## Supplementary Information

**Additional file 1.**

## Data Availability

The datasets used and/or analyzed during the current study available from the corresponding author on reasonable request.
