# Supplementary material for: The impact of training healthcare professionals’ communication skills on the clinical care of diabetes and hypertension: a systematic review and meta-analysis
Source: BMC Fam Pract. 2021 Jul 15;22:152. doi: 10.1186/s12875-021-01504-x (PMC8281627; doi:10.1186/s12875-021-01504-x)
Supplement: Supplementary file 1 — Additional file 1. [file 12875_2021_1504_MOESM1_ESM.docx]

**Supplementary file 1**

1. **Search strategy for Ovid MEDLINE**

1 physician*.mp.

2 doctor*.mp.

3 clinician*.mp.

4 General Practitioner*.mp.

5 specialis*.mp.

6 nurse*.mp.

7 pharmacist*.mp.

8 health professional*.mp.

9 health provider*.mp.

10 1 or 2 or 3 or 4 or 5 or 6 or 7 or 8 or 9

11 exp Communication/

12 communicat*.mp.

13 consultation*.mp.

14 conversation*.mp.

15 interview.mp.

16 shared decision making.mp.

17 11 or 12 or 13 or 14 or 15 or 16

18 train*.mp.

19 course*.mp.

20 program*.mp.

21 intervention*.mp.

22 workshop*.mp.

23 teach*.mp.

24 learn*.mp.

25 educat*.mp.

26 module*.mp.

27 session*.mp.

28 curriculum*.mp.

29 18 or 19 or 20 or 21 or 22 or 23 or 24 or 25 or 26 or 27 or 28

30 exp Diabetes Mellitus/

31 diabet*.mp.

32 (IDDM or NIDDM or MODY or T1DM or T2DM or T1D or T2D).mp.

33 (non insulin* depend* or non insulin* depend* or non insulin?depend* or non insulin?depend*).mp.

34 (insulin* depend* or insulin?depend*).mp.

35 hypertension.mp.

36 hypertens$.mp.

37 blood pressure.mp.

38 30 or 31 or 32 or 33 or 34 or 35 or 36 or 37

39 randomized controlled trial.pt.

40 controlled clinical trial.pt.

41 randomized.ab.

42 placebo.ab.

43 drug therapy.fs.

44 randomly.ab.

45 trial.ab.

46 groups.ab.

47 39 or 40 or 41 or 42 or 43 or 44 or 45 or 46

48 exp animals/ not humans/

49 47 not 48

50 10 and 17 and 29 and 38 and 49

Note: RCT filters were referenced to Higgins JPT, Thomas J, Chandler J, Cumpston M, Li T, Page MJ, Welch VA (editors). Cochrane Handbook for Systematic Reviews of Interventions version 6.2 (updated February 2021). Cochrane, 2021. Available from [www.training.cochrane.org/handbook](http://www.training.cochrane.org/handbook).

1. **Search strategy for Embase**

1 physician*.mp.

2 doctor*.mp.

3 clinician*.mp.

4 General Practitioner*.mp.

5 specialis*.mp.

6 nurse*.mp.

7 pharmacist*.mp.

8 health professional*.mp.

9 health provider*.mp.

10 1 or 2 or 3 or 4 or 5 or 6 or 7 or 8 or 9

11 exp Communication/

12 communicat*.mp.

13 consultation*.mp.

14 conversation*.mp.

15 interview.mp.

16 shared decision making.mp.

17 11 or 12 or 13 or 14 or 15 or 16

18 train*.mp.

19 course*.mp.

20 program*.mp.

21 intervention*.mp.

22 workshop*.mp.

23 teach*.mp.

24 learn*.mp.

25 educat*.mp.

26 module*.mp.

27 session*.mp.

28 curriculum*.mp.

29 18 or 19 or 20 or 21 or 22 or 23 or 24 or 25 or 26 or 27 or 28

30 exp Diabetes Mellitus/

31 diabet*.mp.

32 (IDDM or NIDDM or MODY or T1DM or T2DM or T1D or T2D).mp.

33 (non insulin* depend* or non insulin* depend* or non insulin?depend* or non insulin?depend*).mp.

34 (insulin* depend* or insulin?depend*).mp.

35 hypertension.mp.

36 hypertens$.mp.

37 blood pressure.mp.

38 30 or 31 or 32 or 33 or 34 or 35 or 36 or 37

39 Randomized controlled trial/

40 Controlled clinical study/

41 random$.ti,ab.

42 randomization/

43 intermethod comparison/

44 placebo.ti,ab.

45 (compare or compared or comparison).ti.

46 ((evaluated or evaluate or evaluating or assessed or assess) and (compare or compared or comparing or comparison)).ab.

47 (open adj label).ti,ab.

48 ((double or single or doubly or singly) adj (blind or blinded or blindly)).ti,ab.

49 double blind procedure/

50 parallel group$1.ti,ab.

51 (crossover or cross over).ti,ab.

52 ((assign$ or match or matched or allocation) adj5 (alternate or group$1 or intervention$1 or patient$1 or subject$1 or participant$1)).ti,ab.

53 (assigned or allocated).ti,ab.

54 (controlled adj7 (study or design or trial)).ti,ab.

55 (volunteer or volunteers).ti,ab.

56 human experiment/

57 trial.ti. 333158

58 39 or 40 or 41 or 42 or 43 or 44 or 45 or 46 or 47 or 48 or 49 or 50 or 51 or 52 or 53 or 54 or 55 or 56 or 57

59 Cross-sectional study/ not (randomized controlled trial/ or controlled clinical study/ or controlled study/ or randomi?ed controlled.ti,ab. or control group$1.ti,ab.)

60 (((case adj control$) and random$) not randomi?ed controlled).ti,ab.

61 (Systematic review not (trial or study)).ti.

62 (nonrandom$ not random$).ti,ab.

63 Random field$.ti,ab.

64 (random cluster adj3 sampl$).ti,ab.

65 (review.ab. and review.pt.) not trial.ti.

66 we searched.ab. and (review.ti. or review.pt.)

67 update review.ab.

68 (databases adj4 searched).ab.

69 (rat or rats or mouse or mice or swine or porcine or murine or sheep or lambs or pigs or piglets or rabbit or rabbits or cat or cats or dog or dogs or cattle or bovine or monkey or monkeys or trout or marmoset$1).ti. and animal experiment/

70 Animal experiment/ not (human experiment/ or human/)

71 59 or 60 or 61 or 62 or 63 or 64 or 65 or 66 or 67 or 68 or 69 or 70

72 58 not 71

1. 10 and 17 and 29 and 38 and 72

Note: RCT filters were referenced to Higgins JPT, Thomas J, Chandler J, Cumpston M, Li T, Page MJ, Welch VA (editors). Cochrane Handbook for Systematic Reviews of Interventions version 6.2 (updated February 2021). Cochrane, 2021. Available from [www.training.cochrane.org/handbook](http://www.training.cochrane.org/handbook).

1. **Search strategy for CINAHL**

S1 "physician*"

S2 "doctor*"

S3 "clinician*"

S4 "General Practitioner*"

S5 "specialis*"

S6 "nurse*"

S7 "pharmacist*"

S8 "health professional*"

S9 "health provider*"

S10 S1 OR S2 OR S3 OR S4 OR S5 OR S6 OR S7 OR S8 OR S9

S11 (MH "Communication+")

S12 "communicat*"

S13 "consultation*"

S14 "conversation*"

S15 "interview"

S16 "shared decision making"

S17 S11 OR S12 OR S13 OR S14 OR S15 OR S16

S18 "train*"

S19 "course*"

S20 "program*"

S21 "intervention*"

S22 "workshop*"

S23 "teach*"

S24 "learn*"

S25 "educat*"

S26 "module*"

S27 "session*"

S28 "curriculum*"

S29 S18 OR S19 OR S20 OR S21 OR S22 OR S23 OR S24 OR S25 OR S26 OR S27 OR S28

S30 (MH "Diabetes Mellitus+")

S31 "Diabetes Mellitus" OR "diabet*"

S32 "IDDM"

S33 (MH "Diabetes Mellitus, Type 2")

S34 "MODY"

S35 "T1DM"

S36 "T2DM"

S37 "T1D"

S38 "T2D"

S39 "non insulin* depend*"

S40 "non insulin* depend*"

S41 "insulin* depend*"

S42 "hypertension"

S43 "hypertens*"

S44 "blood pressure"

S45 S30 OR S31 OR S32 OR S33 OR S34 OR S35 OR S36 OR S37 OR S38 OR S39 OR S40 OR S41 OR S42 OR S43 OR S44

S46 MH randomized controlled trials

S47 MH double‐blind studies

S48 MH single‐blind studies

S49 MH random assignment

S50 MH pretest‐posttest design

S51 MH cluster sample

S52 TI (randomised OR randomized)

S53 AB (random*)

S54 TI (trial)

S55 MH (sample size) AND AB (assigned OR allocated OR control)

S56 MH (placebos)

S57 PT (randomized controlled trial)

S58 AB (control W5 group)

S59 MH (crossover design) OR MH (comparative studies)

S60 AB (cluster W3 RCT)

S61 MH animals+

S62 MH (animal studies)

S63 TI (animal model*)

S64 S61 OR S62 OR S63

S65 MH (human)

S66 S65 not S64

S67 S46 OR S47 OR S48 OR S49 OR S50 OR S51 OR S52 OR S53 OR S54 OR S55 OR S56 OR S57 OR S58 OR S59 OR S60

S68 S67 not S66

S69 S10 AND S17 AND S29 AND S45 AND S68

Note: RCT filters were referenced to Higgins JPT, Thomas J, Chandler J, Cumpston M, Li T, Page MJ, Welch VA (editors). Cochrane Handbook for Systematic Reviews of Interventions version 6.2 (updated February 2021). Cochrane, 2021. Available from [www.training.cochrane.org/handbook](http://www.training.cochrane.org/handbook).

1. **Search strategy for PsycINFO**

1 physician*.mp.

2 doctor*.mp.

3 clinician*.mp.

4 General Practitioner*.mp.

5 specialis*.mp.

6 nurse*.mp.

7 pharmacist*.mp.

8 health professional*.mp.

9 health provider*.mp.

10 1 or 2 or 3 or 4 or 5 or 6 or 7 or 8 or 9

11 exp Communication/

12 communicat*.mp.

13 consultation*.mp.

14 conversation*.mp.

15 interview.mp.

16 shared decision making.mp.

17 11 or 12 or 13 or 14 or 15 or 16

18 train*.mp.

19 course*.mp.

20 program*.mp.

21 intervention*.mp.

22 workshop*.mp.

23 teach*.mp.

24 learn*.mp.

25 educat*.mp.

26 module*.mp.

27 session*.mp.

28 curriculum*.mp.

29 18 or 19 or 20 or 21 or 22 or 23 or 24 or 25 or 26 or 27 or 28

30 exp Diabetes Mellitus/

31 diabet*.mp.

32 (IDDM or NIDDM or MODY or T1DM or T2DM or T1D or T2D).mp.

33 (non insulin* depend* or non insulin* depend* or non insulin?depend* or non insulin?depend*).mp.

34 (insulin* depend* or insulin?depend*).mp.

35 hypertension.mp.

36 hypertens$.mp.

37 blood pressure.mp.

38 30 or 31 or 32 or 33 or 34 or 35 or 36 or 37

39 random*.mp.

40 control*.mp.

41 exp Treatment/

42 39 or 40 or 41

43 10 and 17 and 29 and 38 and 42

Note: RCT filters were referenced to Eady AM, Wilczynski NL, Haynes RB. PsycINFO search strategies identified methodologically sound therapy studies and review articles for use by clinicians and researchers. Journal of Clinical Epidemiology.  2008 Jan;61(1):34-40.

1. **Search strategy for Cochrane Central Register of Controlled Trials (CENTRAL) and Cochrane Database of Systematic Reviews (CDSR)**

#1 (physician*):ti,ab,kw

#2 (doctor*):ti,ab,kw

#3 (clinician*):ti,ab,kw

#4 (General Practitioner*):ti,ab,kw

#5 (specialis*):ti,ab,kw

#6 (nurse*):ti,ab,kw

#7 (pharmacist*):ti,ab,kw

#8 (health professional*):ti,ab,kw

#9 (health provider*):ti,ab,kw

#10 #1 or #2 or #3 or #4 or #5 or #6 or #7 or #8 or #9

#11 (Communication):ti,ab,kw

#12 (communicat*):ti,ab,kw

#13 (consultation*):ti,ab,kw

#14 (conversation*):ti,ab,kw

#15 (interview):ti,ab,kw

#16 (shared decision making.):ti,ab,kw

#17 #11 or #12 or #13 or #14 or #15 or #16

#18 (train*):ti,ab,kw

#19 (course*):ti,ab,kw

#20 (program*):ti,ab,kw

#21 (intervention*):ti,ab,kw

#22 (workshop*):ti,ab,kw

#23 (teach*):ti,ab,kw

#24 (learn*):ti,ab,kw

#25 (module*):ti,ab,kw

#26 (session*):ti,ab,kw

#27 (curriculum*):ti,ab,kw

#28 (educat*):ti,ab,kw

#29 #18 or #19 or #20 or #21 or #22 or #23 or #24 or #25 or #26 or #27 or #28

#30 (Diabetes Mellitus):ti,ab,kw

#31 (diabet*):ti,ab,kw

#32 ((IDDM or NIDDM or MODY or T1DM or T2DM or T1D or T2D)):ti,ab,kw

#33 ((non insulin* depend* or non insulin* depend* or non insulin?depend* or non insulin?depend*)):ti,ab,kw

#34 ((insulin* depend* or insulin?depend*)):ti,ab,kw

#35 (hypertension):ti,ab,kw

#36 (hypertens$):ti,ab,kw

#37 (blood pressure):ti,ab,kw

#38 #30 or #31 or #32 or #33 or #34 or #35 or #36 or #37

#39 #10 and #17 and #29 and #38

1. **Search strategy for ClinicalTrials.gov and World Health Organization International Clinical Trials Registry Platform**

Condition or disease:

Diabetes or hypertension or blood pressure

Other terms:

Communication or consultation or conversation or interview or shared decision making
